# Severe small intestinal bacterial overgrowth syndrome after jejunal feeding requiring surgical intervention: a case report and review of the literature

**DOI:** 10.1186/s12876-022-02370-2

**Published:** 2022-06-20

**Authors:** Majd B. Aboona, Tina W. Wong, Paul R. Del Prado, Keith Paley, Ross F. Goldberg, Samuel Weimer, Harikrishna Dave, Dan Hobohm, Adam Smith

**Affiliations:** 1 University of Arizona College of Medicine, 475 N 5th St, Phoenix, AZ 85004 USA; 2 Department of Surgery, Creighton University School of Medicine, Phoenix, AZ USA; 3 Department of Surgery, Valleywise Health Medical Center, Phoenix, AZ USA; 4 Department of Pathology, Valleywise Health Medical Center, Phoenix, AZ USA

**Keywords:** Small intestinal overgrowth syndrome, Pneumatosis intestinalis, Portal venous gas, Enteral feeding, Gastric cancer, Gastric outlet obstruction, Immunosuppression

## Abstract

**Background:**

Small intestinal bacterial overgrowth (SIBO) is a condition of unknown prevalence characterized by an excessive amount of bacteria in the small bowel, typically resulting in vague gastrointestinal symptoms with bloating being most commonly reported. Here we describe a severe case of SIBO leading to small bowel necrosis requiring surgical intervention.

**Case presentation:**

A 55-year-old Hispanic female with gastric outlet obstruction secondary to a newly diagnosed gastric adenocarcinoma, receiving neoadjuvant chemotherapy, developed bloody gastrostomy output and rapidly progressing nausea and abdominal distention 3 days after jejunostomy tube placement and initiation of jejunal enteral nutrition. Imaging revealed diffuse pneumatosis and portal venous gas. Surgical exploration confirmed segmental bowel necrosis requiring resection. Histologic findings were consistent with SIBO.

**Conclusions:**

Presentation of severe SIBO in the setting of intestinal stasis secondary to gastric outlet after initiation of enteral feeds is a rare phenomenon. Early recognition and diagnosis of SIBO is critical in minimizing patient morbidity and mortality.

## Background

Small intestinal bacterial overgrowth (SIBO) is a syndrome in which an excessive amount of bacteria is found within the small bowel. The commonly described symptoms of SIBO include bloating, flatulence, nausea, abdominal distension, diarrhea or constipation, and abdominal pain/discomfort. Malabsorption can be seen in severe cases. Due to the nonspecific nature of its clinical manifestations, the true prevalence of SIBO is unknown [1–3]. Physical barriers such as the ileocecal valve and physiologic mechanisms such as small bowel motility, gastric acid, pancreaticobiliary secretions, and systemic and local immunity help to prevent SIBO [4, 5]. The currently accepted standard for diagnosing SIBO is quantitative microbial investigation of duodenal or jejunal aspirates [2, 4]. Breath testing using carbohydrate substrates such as glucose or lactulose provides an accepted alternative non-invasive testing for SIBO. The most recent guidelines have considered > 10^3^ CFU/mL to be diagnostic for SIBO [2]. When diagnosed, the cornerstone of treatment consists of a course of antibiotic therapy directed at aerobic and anaerobic enteric pathogens as SIBO is frequently due to a polymicrobial overgrowth. Recommended antibiotics can include amoxicillin-clavulanic acid, chlortetracycline, ciprofloxacin, doxycycline, metronidazole, neomycin, norfloxacin, rifaximin, tetracycline, and trimethoprim-sulfamethoxazole [2, 6–8]. However, here we describe a severe case of SIBO that led to bowel necrosis and the need for surgical intervention.

## Case presentation

**Fig. 1:**
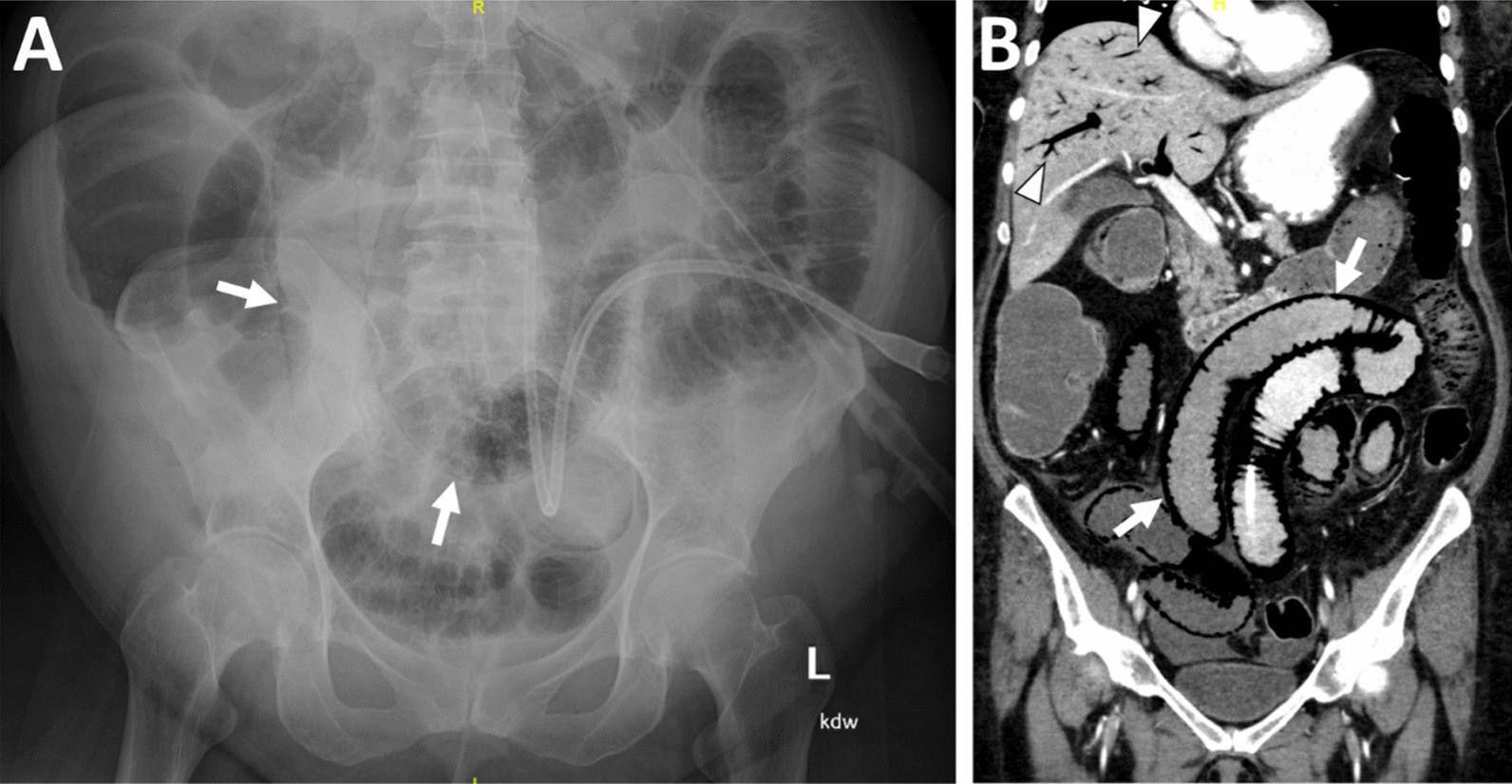
Abdominal X-ray **A** and CT **B** showing pneumatosis intestinalis (arrow) and portal venous gas (arrowhead)

A 55-year-old Hispanic female with newly diagnosed T3N0 poorly differentiated gastric adenocarcinoma with signet ring cell features presented to the Valleywise Health Medical Center Emergency Department 2 weeks after pyloric stenting and first cycle of neoadjuvant chemotherapy at a different facility with symptoms of intractable nausea and emesis. Initial workup with computed tomography (CT) scan was concerning for an occluded stent resulting in gastric outlet obstruction. Patient was admitted to the hospital and underwent an esophagogastroduodenoscopy (EGD) that revealed high grade stricture due to tumor ingrowth within the stent. General Surgery was thus consulted, and a venting gastrostomy tube and distal feeding jejunostomy tube placement was recommended at this time to bridge the patient through her neoadjuvant chemotherapy prior to definitive cancer resection. The surgery was uncomplicated, and patient was initiated on trickle jejunal tubefeeds on postoperative day (POD) 1 and advanced to goal by POD2. Patient initially tolerated the tubefeeds well but was noted with interval development of a diffuse maculopapular rash that improved mildly with antihistamine therapy. On POD3 patient began experiencing abdominal discomfort and nausea, with return of new bloody output from her venting gastrostomy tube. Within a period of 6 h, her abdominal exam became significantly more distended and tender without peritonitis, and the patient became increasingly lethargic. An abdominal plain film at this time revealed dilated loops of small bowel with pneumatosis. A follow up CT scan confirmed presence of diffuse pneumatosis as well as portal venous gas (Fig. 1).

**Fig. 2:**
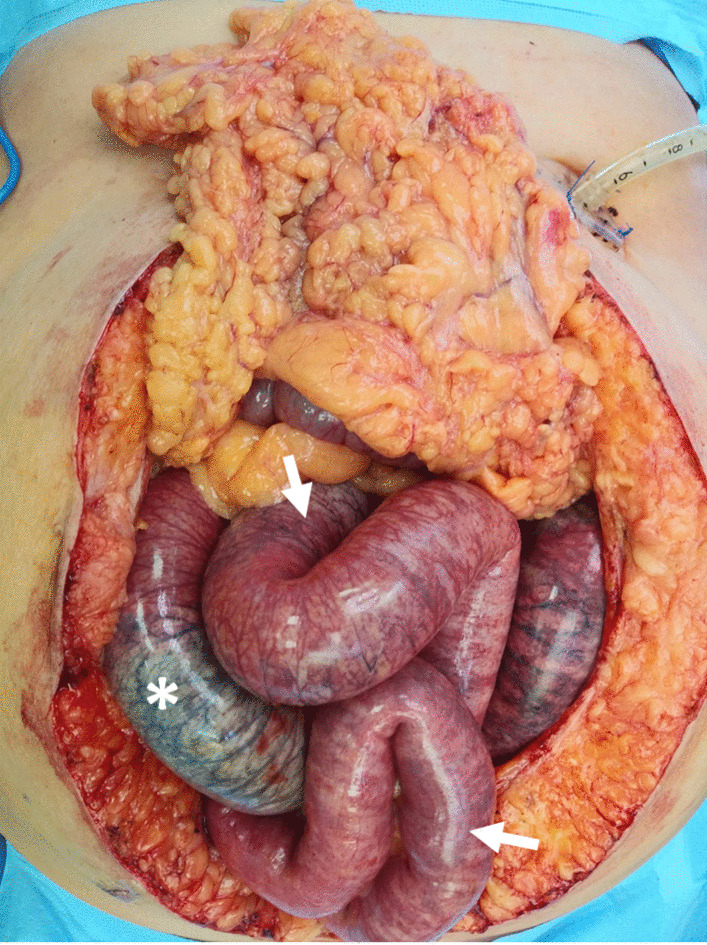
Intraoperative findings from abdominal exploration revealing segments of frankly necrotic small bowel (asterisk) and areas of viable bowel with palpable bowel wall crepitus consistent with pneumatosis intestinalis (arrow)

**Fig. 3:**
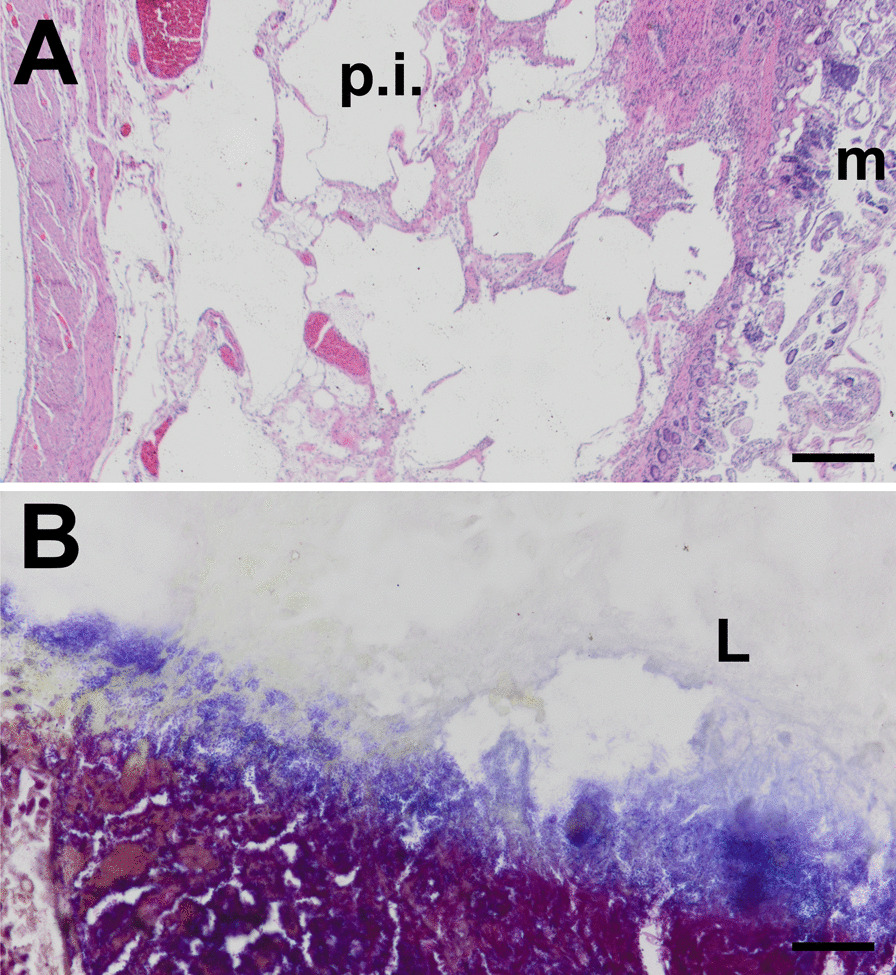
Surgical pathology demonstrates pneumatosis intestinalis and bowel necrosis (**A**; hematoxylin and eosin stain, scale bar = 100 µm) and intraluminal overabundance of microorganisms (**B**; gram stain, scale bar = 10 µm). p.i., pneumatosis intestinalis; m, mucosa; L, lumen. Images were obtained using an Olympus BX51 microscope with 40 × high resolution and 100 × oil immersion using Olympus UIS 2 UPlan FLN objective lenses and captured using an Olympus DP23 camera at a resolution of 96 DPI without downstream processing. Images were captured using the Olympus CellSens acquisition software

The patient was taken emergently for an exploratory laparotomy, and a total of 160 cm of small bowel was resected for frank necrosis (Fig. 2). The bowel was left in discontinuity, and the patient was taken back for two more exploratory laparotomies where more small bowel was resected prior to final restoration of intestinal continuity. A feeding jejunostomy tube was replaced, and jejunal aspirates were collected at this time. Surgical pathology confirmed extensive mucosal necrosis with inflammation, submucosal pneumatosis intestinalis, and foci of transmural inflammation along with an abundance of bacteria with mixed features (Fig. 3). Quantitative cultures from the jejunal aspirates revealed 10^8^ CFU/g. The diagnosis of small intestinal bacterial overgrowth was concluded at this time, and the patient was started on Rifaximin, Zosyn, Bactrim, linezolid, and Diflucan. Serial jejunal aspirates were obtained for quantitative analysis, and the patient was supported nutritionally with total parenteral nutrition for 2 weeks until her bacterial counts were below diagnostic criteria for SIBO to allow for re-initiation of enteral nutrition. Patient was transitioned to PO rifaximin and linezolid, and the tubefeed administration rate was slowly increased to goal over the course of 10 days with careful monitoring of symptoms. Patient was eventually discharged home 7 weeks after initial hospital admission with hospice care and passed away a few months later.

## Discussion and conclusions

SIBO is a condition typically characterized by vague abdominal/gastrointestinal symptoms and managed with medical therapy. However, in this case report, we describe a uniquely severe case of SIBO in which surgical intervention was necessary. There are several conditions within the gastrointestinal tract as well as the patient that can predispose an individual to SIBO.

As its name suggests, the pathophysiology of SIBO relates to an abnormal expansion of bacterial counts within the small intestine. Typically, the duodenum and proximal jejunum contain lactobacilli, enterococci, and gram-positive aerobes or facultative anaerobes that are < 10^4^ CFU/mL [4]. The increase in intestinal microorganisms in SIBO can be a result of direct extension of colonic bacteria into the small bowel. Alternatively, bacteria normally found in the small bowel can increase in number as a result of intestinal dysmotility or relative stasis [2, 4]. These bacteria interfere with the normal gastrointestinal function of the patient. They also contain endotoxins that can result in systemic effects such as release of inflammatory cytokines or in more serious cases lactic acidosis [4]. Anatomical changes to the GI tract such as small intestinal obstruction and stagnation like strictures, tumors, effects from a previous abdominal surgery (e.g. intestinal bypass and blind loop syndrome) can result in SIBO [2]. Physiologic changes to motility in the GI tract such as achlorhydria due to long term administration of PPIs, exocrine pancreatic insufficiency due to lack of antibacterial effect of proteolytic enzymes, and immunodeficiency syndromes can contribute to development of SIBO [2, 4]. Furthermore, intestinal motility can be affected from electrolyte abnormalities such as hypokalemia or hypomagnesemia.

Although this presentation of severe SIBO necessitating surgical intervention has not previously been described in the literature, there have been reports of cases of clinically significant pneumatosis intestinalis in patients receiving tubefeeds [9, 10]. Tubefeed associated bowel necrosis is a rare occurrence with an incidence of 0.15% in a large population, and it is unclear what role tubefeeds may play in the development of intestinal ischemia. Tubefeed intolerance can begin with symptoms of abdominal distension and progress to clinically significant pneumatosis intestinalis, hypotension, and shock as a rare complication if unrecognized and managed appropriately [9, 10].

Our case report is a unique patient presentation of SIBO that developed after initiation of tubefeeds. We hypothesize that gastric outlet obstruction and/or a post-operative ileus resulting in intestinal stasis along with immunosuppression from cancer and chemotherapy predisposed this patient to SIBO. Mild electrolyte derangements in the days preceding the patient’s decompensation may have additionally contributed to the decreased intestinal mobility. Resumption of enteral nutrition in the form of jejunal tubefeeds subsequently exposed the bacteria within the small intestine to a rich nutrient source which led to their overgrowth. It is essential to be cognizant of early signs and symptoms of SIBO in individuals with identifiable risk factors. The patient denied a history of food or drug allergy, thus we suspected the development of diffuse rash 1 day after her jejunostomy tube placement and initiation of tubefeed may have been the sequela of endotoxin release from the bacterial overgrowth and in fact an early sign of SIBO. Other signs and symptoms that subsequently developed in this patient included intractable nausea, emesis, abdominal distension, and bloody gastric output. In addition, this patient developed pneumatosis intestinalis with portal venous gas which prompted emergent surgical intervention. The patient’s immunosuppressed state furthermore contributed to her rapid clinical deterioration given the lack of host response to defend against her abdominal sepsis.

Serial jejunal aspirates helped monitor the progression of disease and responsiveness to treatment in this patient. There is not much discussion in the literature regarding the effectiveness of serial aspirates in individuals with SIBO, which may in part be due to the difficulty of obtaining these samples in patients without direct enteric access. For our patient, the ability to obtain serial aspirates through the jejunostomy tube provided a critical advantage for careful antibiotic management and determination of timing of resolution of SIBO and readiness for resuming enteral feeds.

It is important to recognize that while pneumatosis intestinalis was seen as a surgical emergency decades ago, it is now widely accepted that pneumatosis intestinalis itself in fact can be self-limiting and reversible with appropriate medical management of its underlying cause and therefore does not mandate operative intervention. Similar to treatment of pneumatosis intestinalis seen in neonatal necrotizing enterocolitis (NEC), early bowel rest, intravenous hydration, antibiotic administration, and parenteral nutrition are key components to successful nonoperative management. The progression to peritonitis, portal venous gas, pneumoperitoneum, hemodynamic instability, and shock however are indications of bowel necrosis and intestinal perforation and therefore warrant surgical exploration.

It is unclear if earlier recognition of SIBO would have changed the clinical course in our patient and helped to avoid the repeated laparotomies and extensive bowel resection. Given the rarity of this condition, there is no established guidelines management of these patients with high risk of developing severe SIBO. However, we propose that a slower introduction and up-titrating of tubefeeds along with consideration for administration of prophylactic antibiotics may be help to minimize this risk.

While SIBO typically presents with a variety of vague abdominal symptoms and can be successfully treated on an outpatient basis, here we describe a unique case of severe SIBO in the setting of relative intestinal stasis secondary to gastric outlet obstruction and/or postoperative ileus in an immunosuppressed patient after initiation of enteral feeds. Given the rarity of severe SIBO such as our current case, there is an unsurprising lack of evidence-based clinical guidelines toward its management. However, the understanding of its entity and its pathophysiology can help clinicians in its early recognition and diagnosis in order to minimize patient morbidity and mortality from this unusual condition.

## Data Availability

All data are included in this article and subsequent figures.

## Abbreviations

SIBO: Small intestinal bacterial overgrowth
CT: Computed tomography
EGD: Esophagogastroduodenoscopy
POD: Post operative day
CFU: Colony forming unit
g: Gram
GI: Gastrointestinal
mL: Milliliter
